# Practical Electricity in Medicine and Surgery

**Published:** 1890-05

**Authors:** 


PRACTICAL ELECTRICITY IN MEDICINE AND SURGERY.—By G. A. Liebig, Jr., Ph. D., Assistant in Electricity, John Hopkins University, etc., etc., and Geo. H. Rohe, M. D., Prof. Obstetrics and Hygiene, College of Physicians and Surgeons, Baltimore, Md. F. A. Davis, Publisher, Philadelphia and London. 1890.

---

The growing interest which is manifesting itself daily in the subject of Electro-Therapeutics has led to much search and development in this important branch of medicine. Empirical methods of application have been, to a considerable extent, superseded by rational ones, and the control of therapeutical results has become comparatively certain, when formerly it was purely of a haphazard character.

It is of prime importance to a practical knowledge of electro-therapeutics that one should first be conversant with the fundamental principles of electricity—that is, the varieties, character and direction of currents; principles of production and induction, and the instruments used for its development and application. The work before us meets this requirement, and presents facilities for acquiring this knowledge surpassed by none. It is gotten up with much care, and rare judgment. Its style is neat, and its descriptions clear, explicit and devoid, as much as possible, of confusing technicalities. The learner is gotten by easy degrees into the very pith of the study, and the mind retains the principles the more easily, as they are expressed in plain terms, with no multidæ of obscure technicalities to define and reconcile.

The book is divided into three parts. Part I containing a full and clear description of electricity and magnetism, with the various scientific details and terms connected with each. Part II is devoted to electro-physiological questions and apparatus, while Part III is the more practical part, and treats methods of medical and surgical application.

It is a work that cannot be dispensed with. Learners or beginners particularly, will find it invaluable, and to all who take an interest in electrical questions, we commend these three hundred and eighty odd pages as invaluable adjuncts to their library. R. W. W.

---

### THE THERAPEUTICS OF HAEMOGLOBIN COMPOUND.

The predigestion of foods has done much for the dietary of invalids and convalescents from acute disease or with anæmia and enfeebled digestion.

It must be admitted, however, that many cases require freqently in devitalizing diseases some efficient method of rapid nutrition, capable of ready absorption without taxing the digestive functions, to combat the anæmia.

This is furnished most naturally by the circulating medium itself—blood containing the elements of nutrition in assimilable form—and a preparation of bullock's blood entitled Hæmoglobin Compound has been prepared which seems to meet the indications admirably.

Experiments with this preparation have been in progress by its author, Dr. F. E. Stewart, for ten years past, and Hæmoglobin as now marketed by Parke, Davis & Co., is the result.

This preparation has many advantages as a nutrient stimulant and samples of it and literature descriptive of its application will be furnished physicians, on request.

---

EARACHE.—Take five parts of camphorated chloral, thirty parts of glycerine, and ten parts of the oil of sweet almonds. A piece of cotton is saturated and introduced well into the ear; and it is also rubbed behind the ear. The pain is relieved as if bymagic, and, if there is inflammation, it often subsides quickly.

